# A Case Series of Laparoscopic Colorectal Resections with Natural Orifice Specimen Extraction and Systematic Literature Review

**DOI:** 10.1055/s-0041-1733833

**Published:** 2021-08-03

**Authors:** Nasir Zaheer Ahmad, Ray Swayamjoti, Karen Flashman, Syed Abul Hassan Naqvi, Jim Khan

**Affiliations:** 1 Department of General Surgery, University Hospital Limerick, Limerick, Ireland; 2 Department of Colorectal Surgery, Queen Alexandra Hospital, Portsmouth, United Kingdom

**Keywords:** minimal access surgery, natural orifice specimen extraction, cosmetic

## Abstract

**Background** Minimal access surgery is associated with improved cosmetic and other short-term outcomes. Conventionally, an abdominal incision is made for specimen extraction. We assessed the feasibility of specimen extraction through one of the natural orifices and analyzed its impact on short-term outcomes.

**Methods** A prospectively collected data were reviewed on consecutive patients who underwent natural orifice specimen extraction (NOSE) after laparoscopic colorectal surgery. The results were compared with a matched group who had transabdominal extraction (TAE) of the specimens. A systematic literature review was performed to compare our results.

**Results** The combined median operating time for right and left colectomies was significantly higher in the NOSE group as compared with TAE group (260 vs. 150). There was no mortality in either group and no conversions to TAE in the NOSE group. No local metastasis or major iatrogenic injuries were reported at the time of retrieval. The results were comparable to those of a meta-analysis of randomized controlled trials.

**Conclusion** The results of NOSE are comparable to those of TAEs. The absence of a minilaparotomy for specimen extraction may lead to a speedy recovery and better cosmesis.

It has been more than two decades since the dawn of minimal access surgery for colorectal pathologies. 1 2 In the early years, there was huge skepticism and concern regarding its feasibility and safety, in particular, in dealing with malignant disorders. Some of the landmark publications of the current era have proved not only the feasibility but also the superiority of laparoscopy with regard to the short-term outcomes. 3 4 5 6 The equivalence in oncological outcomes was the driving factor to justify its implementation in clinical practice. 7

With the increasing experience of surgeons in laparoscopy and improvements in technology, there has been an increasing trend on reducing the invasiveness of surgical procedures further. The single incision laparoscopic surgery (SILS) emerged in 2008 to improve the cosmesis and enhance recovery without compromising patient safety and oncological outcomes. 8 Natural orifice transluminal endoscopic surgery (NOTES) was introduced after couple of years to enhance postoperative cosmetic appearance and to combat the wound-related complications. 9 Because of the technical and economic reasons, both NOTES and SILS did not gain enough popularity to become the gold standard in the world of minimal access surgery. Natural orifice specimen extraction (NOSE) after conventional laparoscopic resections was described as a prequel to NOTES and was put forward as an alternative option to achieve the goal of scar less surgery. 10 The NOSE surgery was examined by several researchers for its safety and was reported as a fair and feasible option. 11 12

Most of the evidence supporting NOSE surgery is based on retrospective literature making it hard to recommend the routine use of this novel technique. One of the main constraints in the production of high-quality evidence is probably the learning curve involved in the acquisition of this surgical skill. The aim of this case series was to compare the safety and feasibility of NOSE with transabdominal extraction (TAE) and to compare the results with the available evidence.

## Methods

A prospective database was reviewed for all patients who underwent elective laparoscopic colorectal resections with NOSE for benign and malignant colorectal pathologies. The number of patients who underwent hybrid laparoscopy with NOSE was 35 and they were all performed by two experienced surgeons in a high-volume cancer research center after local clinical governance approval and after undertaking informed patient consent. A matched group of patients who had TAE of specimen after colorectal surgery during the same period was selected for comparison. These TAE patients were 1:1, near neighbor, propensity-score matched to the above-mentioned 35 consecutive NOSE patients using age, gender, American Society of Anesthesiologists (ASA), and disease characteristics (benign/malignant) as covariates in the linear regression with method of extraction as dependent variable to calculate the propensity scores.

Elective patients with benign or malignant disease between the age of 18 and 80 years with ASA score of less than III and body mass index (BMI) of less than 35 were included in this study. Among the malignant cases, a tumor size of less than 4 cm and a T stage of less than T3 with no neoadjuvant therapy were considered suitable for inclusion. The malignant cases or nonendoscopically resectable polyps had an upper limit of the tumor size of 4 cm in this series.

The benign cases included ulcerative colitis, Crohn's disease, diverticulosis, and endometriosis. The decision of NOSE versus TAE was made after explicit patient consent and the suitability of the patient and surgeon preference, and BMI did not influence the decision-making. In the females with right hemicolectomy, the NOSE approach was performed with transvaginal extraction and in the females with TAE, it was the midline extraction.

Patients presenting with inflammatory masses or requiring emergency surgery were excluded from the analysis. The end points studied included operating time, length of hospital stay, conversion rate, reoperation within 30 days, and extraction site complications. A systematic literature review was performed for randomized controlled trials (RCTs) comparing NOSE surgery with conventional TAE of specimen. The results of this case series were compared with those of the meta-analysis.

Student's *t* -test was used to compare parametric data. The parametric and nonparametric data were analyzed using Student's *t* -test and two sample Wilcoxon rank test, respectively. Chi-square test was used for analysis of categorical variables. A p-value of less than 0.05 was considered significant and reported accordingly. Nonsignificant differences were also reported. Calculations were done using STATA version 16.0 and Comprehensive Meta-Analysis version 2 was used for the meta-analysis of the data reported in the literature.

## Surgical Technique

All patients in the NOSE group underwent examination under anesthesia at the time of index operation to rule out vaginal stenosis, congenital abnormalities, or any other variation that would hinder transvaginal or transanal specimen extraction.

All the procedures were performed in modified Lloyd-Davies position on antislip bean bag. Bowel preparation either in the form of two phosphate enema or Picolax was given only for left-sided procedures.

A standard four-port technique was used for laparoscopic right or left hemicolectomy. In conventional surgery, the extraction site was fashioned at transumbilical or suprapubic region measuring approximately 5 to 7 cm. Wound protector was used to prevent any contamination and the tumor coming in direct contact with the open wound to prevent local recurrence. In NOSE surgery, two different methods of specimen extraction were practiced which included the transvaginal and transrectal routes. In female patients, both approaches were adopted. A wound protector was used in the vagina to prevent contamination or tumor cell implantation.

## Transvaginal

In addition to abdomen, vaginal preparations were also performed. The operation set up was similar to laparoscopic colorectal resection. The anastomosis was performed intracorporeally using either a linear Endo GIA or a circular stapler depending on the procedure. At the end of the colonic mobilization, the mesentery of the colon was divided intracorporeally using an energy device and the specimen was liberated free by using a linear cutter Endo GIA stapler. To expose the posterior fornix of vagina, uterus was hitched using a heavy Prolene stitch. A posterior colpotomy was fashioned using diathermy and once adequate size incision was created, a wound protector was inserted to stretch the vaginal wall. A big swab was placed in the introitus to prevent escape of gas from the vaginal wall, and the specimen was then pulled through the vagina carefully using a combination of pull from below and pushes from the abdomen with the help of the assistant. The wound protector was removed and the colpotomy vaginal defect was closed in two layers using absorbable stiches.

## Transanal

A standard laparoscopic colorectal procedure was performed. The bowel was divided below the tumor using Endo GIA stapler. The anal canal was then prepared using antiseptic solution and was irrigated. The proximal division of the colon was performed intracorporeally with a noncrushing instrument placed to prevent spillage. After this point, the rectal stump was opened up using two stay sutures. An extraction bag was inserted through the anal canal. The specimen was placed in the bag and pulled out of the anal canal. Particular care was taken not to stretch the sphincters in order to prevent any sphincter damage. The anvil of the circular stapler was inserted transanally and positioned in the proximal colon. The rectal stump was closed again using Endo GIA stapler. An intracorporeal purse string suture was performed in the proximal site and the circular stapler was inserted through the anal canal to complete the colorectal anastomosis.

## Results

There were 35 patients in this series that underwent laparoscopic surgery with NOSE and a matched group of 35 patients who had TAE of the specimen. The details of patient characteristics and the procedures performed in both groups are given in Tables 1 and 2 .

**Table 1:** Patient characteristics

Variable	NOSE (35)	TAE (35)	Statistical difference
Age, median (range)	59 (29–79)	63 (18–80)	NS
Gender	Male 13, female 22	Male 14, female 21	NS
ASA			
I	7	9	NS
II	23	21	NS
III	5	5	NS
BMI	25.7 (20–33)	25.8 (20–29)	NS
Benign	15	16	NS
Malignant	20	19	NS

Abbreviations: ASA, American Society of Anesthesiologists; BMI, body mass index; NOSE, natural orifice specimen extraction; NS, not significant; TAE, transabdominal extraction.

**Table 2:** Types of operation in each group

Type of operation	NOSE	TAE	Total
Right hemicolectomy	3	21	24
Ileocolic resection	0	1	1
Redo ileocolic resection	0	2	2
Panproctocolectomy	12	0	12
Proctocolectomy	4	0	4
Proctectomy	2	0	2
Left hemicolectomy	0	2	2
Sigmoid colectomy/high anterior resection	14	9	23
Total	35	35	70

Abbreviations: NOSE, natural orifice specimen extraction; TAE, transabdominal extraction.

The median length of small bowel extracted through the natural orifices and the transabdominal approach were 60 (40–165) and 65 (45–180) mm, respectively. The respective lengths of large bowel extracted through these routes were 170 (90–225) and 175 (80–350) mm.

All the operations were performed by colorectal surgeons with an experience of more than 100 laparoscopic resections. Both transvaginal and transrectal extraction of specimen were performed with no conversion to open surgery or TAE in the NOSE group.

The operating time was understandably longer in the NOSE group as compared with the TAE group (260 vs. 150 minutes). The average length of hospital stay was also found to be longer in the NOSE group (5.5 vs. 4 days). Two patients in each group developed minor complications which were managed conservatively. Some major complications requiring intervention were also encountered in both groups. In the NOSE group, one of the patients after sigmoid colectomy developed pelvic collection postoperatively which was managed conservatively with antibiotics and did not require drainage and another patient required reoperation for stoma prolapse. Two patients in the TAE group required reoperation for bleeding and anastomotic leak. There was no extraction site complication or mortality in this series ( Table 3 ).

**Table 3:** Details of study outcomes

Variable	NOSE	TAE	*p* -Value
Average operating time (min)	260	150	<0.001
Right-sided resections (min)	160	180	0.067
Left-sided resections (min)	275	135	0.04
Conversions	0	0	NS
Reoperation	2	3	NS
Complications	2	2	NS
Mortality	0	0	NS
Hospital stay (d)	5.5	4	0.023
Extraction site complications	0	0	NS
Local metastases	0	0	NS

Abbreviations: NOSE, natural orifice specimen extraction; NS, not significant; TAE, transabdominal extraction.

A literature search of Medline, Embase, and Cochrane database for “NOSE” revealed 141, 155, and 5 publications, respectively. A title and abstract screening with exclusion of case reports, case series, retrospective studies, reviews, nonrandomized studies, and duplicate studies identified only two RCTs suitable for a meta-analysis. 13 14 The characteristics of these studies are given in Table 4 . The quantitative analysis was done for the purpose of discussion and comparison with this study ( Table 5 ).

**Table 4:** Characteristics of trials included in meta-analysis

Study	Year	Type	Patients		Indications		BMI		ASA			Age	
			NOSE	TAE	Benign	Cancer	NOSE	TAE	I	II	III	NOSE	TAE
Wolthuis et al	2015	RCT	20	20	30	10	23.5	24	11	29	0	54	58
Leung et al	2013	RCT	35	35	5	65	NG	NG	NG	NG	NG	62	72

Abbreviations: ASA, American Society of Anesthesiologists; BMI, body mass index; NG, not given; NOSE, natural orifice specimen extraction; RCT, randomized controlled trial; TAE, transabdominal extraction.

**Table 5:** Meta-analysis of randomized studies

Outcomes	WMD/OR	95% confidence interval		*p* -Value	*N*	Favors
		Lower	Upper			
Operation time	10.666	0.523	20.808	0.039	2	TAE
Complications	0.553	0.124	2.472	0.438	2	None
Hospital stay	0.000	−0.708	0.708	1.000	2	None
Pain score	−1.159	−1.598	−0.721	0.001	2	NOSE

Abbreviations: *N* , number of studies; NOSE, natural orifice specimen extraction; OR, odds ratio; TAE, transabdominal extraction; WMD, weighted mean difference.

## Discussion

NOSE was earlier described in conjunction with NOTES for gallbladder surgery. 15 The technique evolved further with publications of successful transvaginal and transoral extraction of gastric specimens. 16 17 It was not limited to gastrointestinal tract and encompassed the genitourinary system with some successful reporting of transvaginal extractions after multiport nephrectomy. 18

The concept and feasibility of NOSE have been reported in systematic reviews and meta-analysis in the past. 19 20 21 The evidence supporting the implementation of NOSE technique is somewhat weak primarily because of the retrospective studies included in these systematic reviews. We performed a literature search on the subject in accordance with the Preferred Reporting Items for Systematic Reviews and Meta-Analyses statement guidelines 22 and included RCTs only to calculate the effect size for different end points after NOSE and TEA surgery ( Table 5 ).

It was predicted that NOSE surgery would cause lesser postoperative pain because of the absence of a minilaparotomy for specimen extraction and would speed up the recovery and lead to an early discharge. In this series, however, the hospital stay was relatively longer in the NOSE group as compared with the TAE group. One possible explanation is probably a higher number of right-sided resections in the TAE group and more left-sided resections in NOSE group. A meta-analysis of the RCTs did not show any difference in the length of hospital stay between the two groups.

The average operating time in this case series was longer as compared with the most recent publication by the Belgium Colorectal Group. 14 An average of 260 minutes for NOSE surgery as compared with 150 minutes for TAE may act as a constraint and discourage surgeons from adapting this technique in their routine practice. We believe that extra time taken in NOSE procedures is because of the learning curve that the surgeons are still going through and it is anticipated that over the time they would become more efficient in the technique and would require less time. We hope to see a gradual improvement in the operating time after mastering the essential surgical steps and implementation of the technique in routine practice.

Because of the learning curve issues, we did not attempt the low rectal cancers with NOSE as the anastomosis was stapled and we did not prefer a handsewn anastomosis in these patients. Therefore, the height of the rectal stump mattered as in the early series we wanted to take on high rectal and sigmoid cancers only. The meta-analysis of the randomized trials understandably favored the conventional TAE in this regard ( *p* = 0.039).

Less postoperative pain and minimal analgesia requirement after laparoscopic surgery combined with NOSE have been reported in several studies. Postoperative pain was not assessed formally in this study and the analgesia requirements were not recorded in our database. A TAE or a minilaparotomy for specimen extraction is a potential source of postoperative pain and may increase the risk of wound infection or a future incisional hernia. 23 24 NOSE, on the contrary, minimizes these risks at the cost of a longer operating time. It is believed that operating time would gradually approach to that of TAE in high-volume centers where NOSE is already being practiced. The postoperative pain scores assessed in the meta-analysis of RCTs showed a significant difference in favor of the NOSE technique ( *p* = 0.001).

There was no mortality or life-threatening complication related either to the NOSE surgery or to TAE in this cohort. There were comparable minor and major postoperative complications in both groups that were managed either conservatively or where necessary treated with appropriate interventions. A meta-analysis of randomized trials did not show a significant difference in the rate of complications between NOSE and conventional TAE ( *p* = 0.438). This was in contradiction to the previously observed lower complication rate with NOSE surgery. 25

The NOSE was introduced to improve the cosmesis. Postoperative cosmetic outcome has been assessed in some observational studies and was found to be superior after NOSE technique. 26 27 The cosmetic issues related to NOSE surgery were not analyzed in this cohort; however, one of the randomized trial included in this meta-analysis reported higher cosmetic scores after NOSE surgery.

The NOSE surgery involves intracorporeal anastomosis which is a potential source of bacterial contamination. NOSE surgery has been reported to be associated with bacterial contamination of the peritoneal cavity. 28 No intraoperative bacteriological cultures were taken in this study to assess the extent of contamination after intracorporeal anastomosis. Because of the lack of data, no meta-analysis was performed to find out which technique is responsible for more contamination. However, previous studies have documented more contamination with laparotomy rather than laparoscopy. The authors of this report have been practicing intracorporeal anastomosis for many years and it is observed that the risk of intra-abdominal sepsis secondary to contamination of intracorporeal anastomosis is almost nonexistent.

There were no conversions to conventional TAE in this study and no major complications related to the extraction site were encountered in the NOSE group. The average hospital stay after the surgery was reasonably short and there was no postoperative mortality. There is little doubt on the feasibility and safety of NOSE in research settings; however, on the basis of this study and the literature review, it is not entirely possible to recommend it in routine clinical practice yet. The retrospective design, a small number of patients, a lack of standardization in data collection, and short follow-ups are the limitations of this study. A systematic comparison was done to supplement the discussion on important variables which in itself is somewhat flawed because of the limited number of randomized studies, absence of a formal quality assessment of the studies included in the meta-analysis, and possibility of publication bias. It is evident that there is a definite need for well- powered RCTs on this subject before it can be recommended as a standard of practice.
